# Unveiling Extramedullary Myeloma Immune Microenvironment: A Systematic Review

**DOI:** 10.3390/cancers17071081

**Published:** 2025-03-24

**Authors:** Kassiani Boulogeorgou, Maria Papaioannou, Sofia Chatzileontiadou, Elisavet Georgiou, Amalia Fola, Sofia-Eleni Tzorakoleftheraki, Evdoxia Hatjiharissi, Triantafyllia Koletsa

**Affiliations:** 1 Department of Pathology, School of Medicine, Aristotle University of Thessaloniki, University Campus, 54124 Thessaloniki, Greece; kboulog@auth.gr (K.B.); sofialenatzo@yahoo.com (S.-E.T.); 2 Hematology Unit, 1st Department of Internal Medicine, Aristotle University of Thessaloniki, 54124 Thessaloniki, Greece; papaioam@auth.gr (M.P.); sofia.chatzileontiadou@gmail.com (S.C.); amalia.fola@gmail.com (A.F.); ehatjiharissi@gmail.com (E.H.); 3 Department of Biological Chemistry, School of Medicine, Aristotle University of Thessaloniki, 54124 Thessaloniki, Greece; elgeorgiou@auth.gr

**Keywords:** extramedullary multiple myeloma, plasma cell neoplasm, microenvironment, immune exhaustion, microenvironment heterogeneity

## Abstract

Background/Objectives: In recent years, efforts by the scientific community to elucidate the underlying mechanisms of clonal expansion and selection within tumors have led to the theory of “tumor ecosystems”, implicating, among other factors, the role of the microenvironment in therapy resistance and tumor progression. In this context, the contribution of the microenvironment in the development of multiple myeloma (MM) is being investigated, imparting great emphasis on continuous clonal evolution. This process gives rise to aggressive clones with the potential to spread to extramedullary sites, rendering any treatment strategy practically ineffective. This systematic review aimed to gather knowledge about the immune microenvironment (IME) of extramedullary plasma cell myeloma and the differences in immune synthesis between medullary and extramedullary disease (EMD). Methods: A search strategy according to PRISMA guidelines was conducted in seven databases, and six articles meeting the inclusion criteria were encompassed in the study. Results: Results obtained from molecular analysis as well as flow cytometry and immunofluorescence indicated profound genetic instability at EMD sites along with spatial and temporal heterogeneity of the IME, implying a possible correlation between them. Both genetic and microenvironment variability were notably greater in EMD compared to medullary disease. The establishment of an immunosuppressive microenvironment was the rule, with exhausted CD8+ and natural killer (NK) cells, M2 macrophages, and inactivated dendritic cells found co-localized with neoplastic plasma cells, whereas cytotoxic CD8+ cells, M1 macrophages, and active dendritic cells congregated in tumor-free areas. Post-therapy alterations in the immune milieu were also noted and were concerned mostly the percentages of Tregs and MDSCs. Conclusions: The recognition of the microenvironment-myeloma cell interplay is essential for designing specific therapeutic strategies and ameliorating disease prognosis.

## Simple Summary

Extramedullary disease (EMD) is an advanced-stage disease and is typically characterized by poor clinical outcome. Numerous therapeutic strategies have been applied at times, with insufficient results so far. The current systematic review investigates the composition of the immune microenvironment (IME) at EMD sites and its possible differences between medullary and extramedullary milieu. Six studies meeting our inclusion criteria were analyzed and highlighted the establishment of an immunosuppressive state across the extramedullary niche. A profound spatial and temporal heterogeneity of IME was reported and possibly correlated with defined genetic instability at EMD. Differences were noted between medullary disease and EMD concerning both molecular and microenvironment findings. IME synthesis endured also post-therapy alterations concerning mostly the percentages of T-regulatory cells (Tregs) and Myeloid-derived suppressor cells (MDSCs). Grasping the heterogeneity of IME provides strategic avenues for tailored therapeutic options in EMD.

## 1. Introduction

Extramedullary plasma cell myeloma (EPM) is a rare manifestation of multiple myeloma characterized by the migration of neoplastic cells outside the bone marrow to other tissues or organs. Liver and lymph nodes constitute the most commonly affected sites, whereas breast, skin, neck, gastrointestinal and genitourinary tract are rarely involved [1]. Its incidence varies considerably due to the diagnostic strategy in use [2]. In general terms, extramedullary involvement affects 10–20% of patients with multiple myeloma, mostly those in advanced stages of the disease. The intricate processes causing this extramedullary dissemination are believed to include alterations to adhesion molecules, signaling pathways, and interactions with the bone marrow microenvironment [3,4].

The role of the microenvironment in solid tumors has been underscored in the recent literature. Accordingly, there is a similar great interest in the microenvironment of multiple myeloma (MM). Emerging data highlight the role of the immune microenvironment (IME) in the progression of plasma cell neoplasm [5]. It seems that an immune-altered bone marrow (BM) landscape promotes the advancement of the disease and its resistance to therapies [6]. All the compartments that constitute the multiple myeloma microenvironment in the BM promote immunosuppression and facilitate the survival of MM cells [6].

When neoplastic plasma cells extravasate into extramedullary sites, they form their niche to provide factors that support their survival and growth. The recruited immune cells are usually impaired, leading to an inability to mount effective anti-tumor responses [7]. EPM is often characterized by an aggressive clinical course and poor survival. Hence, there is an unmet need to develop better therapeutic choices for EPM. Reprogramming the immune system to recognize and attack malignant plasma cells could be an attractive treatment option. Therefore, deeper investigation and understanding of both BM and extramedullary IMEs are crucial. In this context, we aimed to conduct a systematic review of original studies in order to gather knowledge primarily on the extramedullary IME and secondarily on its differences to that of bone marrow niche.

## 2. Material and Methods

### 2.1. Search Strategy

A review of the literature was conducted in agreement with the recommendations of Preferred Reporting Items for Systematic Reviews and Meta-Analyses (PRISMA) guidelines [8]. We verified that this particular systematic review was PRISMA-compliant by applying PRISMA 2020 checklist [9]. All research was performed using a pre-specified protocol registered in the open science framework (OSF) database “registration number: https://osf.io/6rg2p/ (accessed on 2 November 2024)”.

### 2.2. Study Selection and Eligibility Criteria

Relevant studies were identified by performing a systematic search in PubMed, Scopus, TRIP, National Library of Medicine, Cochrane Library, MedLinePlus and ClinicalTrials.gov electronic databases. Report examination was conducted until 30 November 2024. As keywords we used “extramedullary multiple myeloma” AND “microenvironment”. The term “immune microenvironment” was not applied in search terms in order to enhance the number of results and avoid the omission of important studies.

Our analysis encompassed all clinical trials, randomized controlled trials, classical articles and meta-analyses written in English language. There was no restriction on the year of publication for search databases in total. No limits were imposed on countries or observation periods. Results of the electronic research from the databases were screened by two authors (KB, TK) independently and selected based on predefined inclusion and exclusion criteria. Specifically, studies were eligible if they were oriented to (a) the synthesis and distribution of the immune microenvironment in extramedullary sites or (b) the differences in immune cell composition among BM and extramedullary localization, (c) the impact of plasma cell myeloma microenvironment in disease progression, and (d) performed on human tissue. On the other hand, exclusion criteria included (a) certain type of documents (abstracts, conferences, reviews, commentaries, editorials), (b) experimental animal or in vitro studies as well as (c) studies referred to clinical diagnosis and treatment of patients with MM and hematological malignancies in general, or (d) to molecular and immunohistochemical profile of neoplastic plasma cells without any mention on tumor microenvironment.

### 2.3. Data Extraction

Each author reviewed the abstracts of the selected articles independently and generated a list of studies to retrieve for full-text review. The lists were compared, and discrepancies were resolved by consensus. Afterwards, the papers were thoroughly studied by both authors and collected data were exported to an Excel table. The recorded information included the following: name of the first author, publication year, doi of the publication, study design, site of EPM, main results focusing on the microenvironment in plasma cell neoplasms as well as differences in neoplastic plasma cells and microenvironment synthesis among bone marrow niche and extramedullary sites.

### 2.4. Quality Assessment (Study Risk-of-Bias Assessment)

The assessment of eligibility of the extracted data was estimated using the Risk-of-Bias In Non-randomized Studies—of Exposure (ROBINS-E) tool [10]. All authors answered the questionnaire of ROBINS-E tool for each article separately and any disagreement was resolved by consensus. The risk-of-bias assessment summary table is illustrated in Supplementary Figure S1.

### 2.5. Primary and Secondary Outcomes

Primary outcomes of this study were the associations of EPM with specific immune microenvironment. Secondary outcomes included differences in the spatial distribution of immune cells, as well as differences between medullary and extramedullary “myeloma niches”.

## 3. Results

### 3.1. Database Search Results

**Figure 1:**
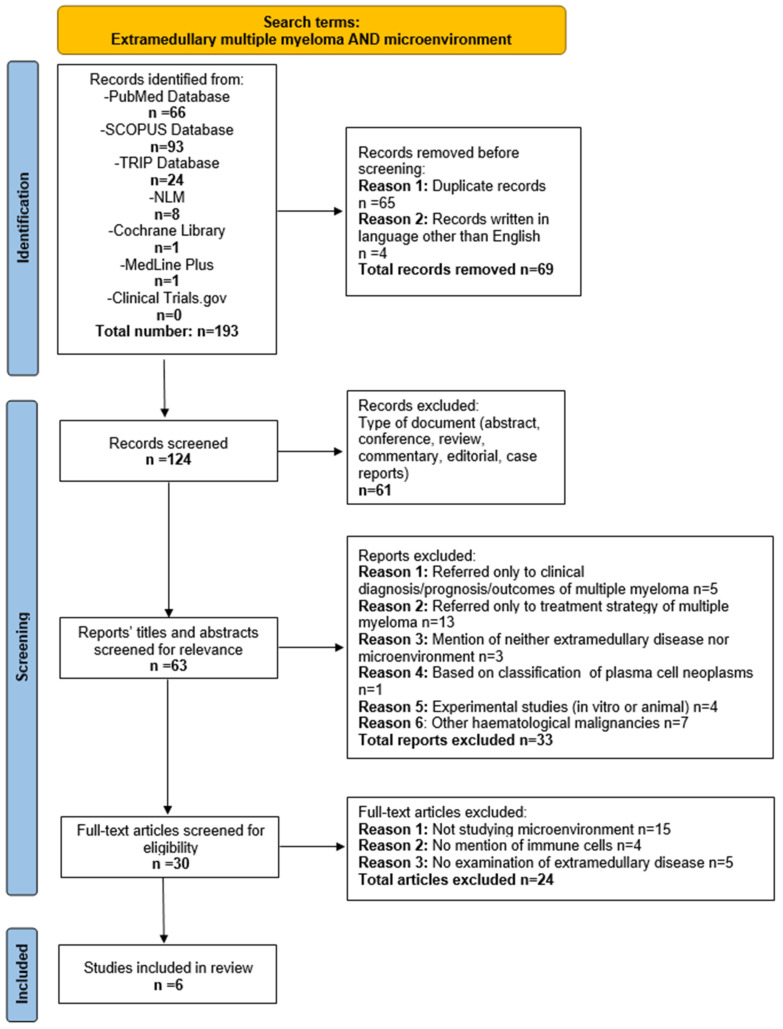
Flow diagram of study selection process.

In total, 193 publications were retrieved from seven databases. Four out of them were excluded since they were written in languages other than English (2 in Czech, 1 in French, and 1 in Japanese). In total, 65 duplicate articles, 47 reviews, 7 case reports, 3 conference abstracts, 2 commentaries, and 2 editorials were removed. Subsequently, 62 articles were screened based on their title and abstract. According to the predefined inclusion and exclusion criteria, 30 reports were assessed through full-text analysis. Of these, 15 were ruled out for not studying the microenvironment at all, 4 studies did not address the IME, and other 5 examined the IME strictly in bone marrow. Finally, six articles that met all the predefined criteria were included in this systematic review. Notably, all the incorporated articles were recent, published within the last five years, (from 2020 to 2024). The study selection process is summarized in Figure 1.

### 3.2. Study Characteristics

The six selected studies included a total of 74 patients with extramedullary disease (EMD), from whom 59 specimens were obtained. Male-to-female ratio was estimated at almost 2:1, comprising 47 males and 27 females. The median age of patients was reported in five studies and was estimated to 55 years with a range from 51 to 61 years [11,12,13,14,15]. In all but one study, samples were obtained through imaging-guided tissue biopsies [11,12,13,15,16]. In the research study carried out by Ryu D. et al., specimens were retrieved by aspiration of ascitic fluid or pleural effusion [14]. Jelinek et al. designated skin as the most frequent site of extramedullary involvement (42.8%) [11], while in the study of Qi et al., EMD concerned primarily breast and liver/spleen (38.8%) [13]. Considering bone-related extramedullary disease, clavicular lesions represented the highest proportion (66.6%), followed by paravertebral (33.3%) [16]. As indicated by Ryu D., Qi Y., and John M, IgG (15/40) was the most prevalent M protein type in EMD specimens, followed by IgA (7/40) [12,13,14]. Pertinent data are displayed in Table 1.

### 3.3. Methodology Used for MM and EPM

To elucidate the cellular composition and spatial distribution of MM and immune cells as well as to define the genomic profile of malignant plasma cells in both intra- and extramedullary sites, molecular analysis was conducted in four out of the six studies utilizing spatial transcriptomics, single-cell RNA sequencing (scRNA-seq), fluorescent in situ hybridization (FISH), and Whole Exome Sequencing (WES) [11,12,14,16]. To further investigate and quantify specific surface markers and target biomolecules in neoplastic or immune cells, flow cytometry and immunofluorescence analysis were performed in four studies, as well [11,13,15,16].

Specifically, Ryu et al. applied scRNA-seq for the simultaneous analysis of myeloma cells and IME in bone marrow and extramedullary sites [14]. scRNA-seq was also the selection strategy method for John et al., who additionally used 10xVisium spatial transcriptomics analysis in order to reveal intratumor heterogeneity in cellular subpopulations within EMD lesions [12]. In the study by Mertz et al., examination of neoplastic cell compartment was accomplished by immunofluorescence, FISH, and WES. The same strategy was subsequently applied for the investigation of the IME. However, in the aforementioned case, to define differences in TCR repertoires between bone marrow and extramedullary lesions, TCR sequencing was also employed [16]. In respect of Jelinek’s comprehensive study, molecular profile of myeloma cells (chromosomal aberrations and mutations in signaling pathways) was detected by FISH and WES. On the other hand, the composition of IME in EPM sites was designated by scRNA-seq and confirmed by bulk RNA-seq and flow cytometry data [11]. A distinct strategy was adopted by Zhang et al. employing multicolored fluorescent flow cytometry to characterize both myeloma and immune cells and to further classify CD3+/CD8+ T-cells into different subsets [15]. Finally, Qi et al. focused solely on the identification of the immunological milieu of pretherapy EMD by utilizing multiplex immunofluorescence [13].

### 3.4. Comparisons Between the Medullary and Extramedullary Disease from a Genetic Perspective

Complete genetic analysis showed no significant differences in the number of mutations between bone marrow and extramedullary lesions. However, remarkable differences were noted in terms of copy number variations, including chromosomes with great prognostic implications such as 1q amplification and 17p deletion [16]. From another perspective, neoplastic cells in extramedullary sites endured activating transcriptional alterations in genes associated with proliferation, glycolysis, proteasome, oxidative phosphorylation, and antigen presentation, while bone marrow cells stimulated the TNFa-induced NF-kB pathway, which intriguingly was found downregulated in most extramedullary cells [14]. Moreover, Jelinek et al. confirmed that EMD characterized by a higher frequency of 1q21 gain, mutations in the MAPK pathway, activation of proliferation-associated pathways and decreased homing to BM [11].

### 3.5. Associations of EPM with Specific Immune Microenvironment

Almost all studies (5/6) confirmed a T-cell exhaustion shift and installation of an immunosuppressive environment in tumor-rich areas [12,13,14,15]. T-cell dysfunction was primarily attributed to the expression of multiple immune-checkpoint receptors (PD-1, LAG-3, TIGIT, TIM-3) on their surface [12,13,15]. Additional potential mechanisms of natural killer (NK) and T-cell inhibition have been unveiled, including regulation of MHC class I or TRAIL gene expression, LIL family protein inactivation, and aberrant expression of HIFA-1 transcription factor [14]. Nevertheless, immunosuppression was not an exclusive privilege of exhausted T- and NK cells. According to Merz et al., myeloid dendritic cells seem to play quite a role while their decline along with an increase in T-regulatory (Treg) and NK cell percentages are implicated in the establishment of an immunosuppressive milieu that contributes to the disease’s ominous progression [16].

In regard to the spatial distribution of immune cells in the tumor microenvironment, CD8+ T-cells with exhausted phenotype (PD-1+, LAG-3+, TIM-3+) and M2 macrophages (CD68+, CD163+, CD86−) were found co-localized with neoplastic plasma cells with an increasing infiltration from “normal” to tumor areas [12,13]. On the contrary, CD8+ cytotoxic T-cells and M1 macrophages (CD68+, CD86+, CD163−) were identified mostly in plasma cell-free areas [12,13]. Intriguing also was the fact that CD58+ dendritic cells (DCs) were detected in tumor-free niches, while the inactivated ones (CD58−) seemed to follow exhausted T-cells in plasma cell-rich niches [12].

Interestingly, when comparing bone marrow niche with extramedullary sites, Merz et al. noticed no significant differences in the immune compartment [16]. In contrast, Zhang et al. concluded that CD3+ T-cell densities were considerably decreased in EMD against BM specimens, in cases of newly diagnosed myeloma [15]. Of note, even though TIGIT and TIM-3 were more frequently expressed on CD8+ T-cells in extramedullary sites compared to bone marrow, no discernible differences were observed regarding their ligand expression, namely CD155 and galectin-9 [15].

### 3.6. Associations of Immune Microenvironment with Therapy and Prognosis

Analysis of microenvironment status after induction therapy with immunomodulatory drugs, proteosome inhibitors, and steroids showed significant enhancement of TCR repertoire interestingly without parallel expansion of distinct TCR clones [16]. Furthermore, the percentages of Tregs and Myeloid-derived suppressor cells (MDSCs) were remarkably higher no matter the degree of treatment response [16]. On the other hand, CAR T-cell therapy with simultaneous targeting of GPRC5D and TNFRSF17 antigens seemed to be quite effective for EMD, succeeding optimal results in cases with a low plasma cell ratio, increased number of M1 macrophages and decreased levels of exhausted CD8 T-cells [12,13]. Comparable were the findings after anti-BCMA therapy where insufficient treatment response was noticed in niches with an abundance of M2 macrophages and inactivated CD8+ cells [13].

## 4. Discussion

Recent research highlights malignant tumors as ecosystems derived from evolving clones impacting the diversity of the microenvironment, which in turn affects tumor progression [17]. In this line, MM is considered a heterogeneous disease, which constitutes several evolving clones with dynamic capabilities [18]. The progression to an aggressive clone can lead to extramedullary dissemination [3], with 6% of multiple myeloma patients exhibiting EMD lesions [12]. The unmet need for effective therapeutic options for these patients possibly persists due to the limited knowledge on the biological mechanisms underlying extramedullary spread. This systematic review emphasizes the insufficient investigation of EMD, with merely two studies comparing the medullary and extramedullary plasma cell populations and microenvironments, despite the increasing evidence underscoring tumor microenvironment as an essential factor for cancer progression [17].

Regarding neoplastic plasma cell population and clonal evolution, cytogenetic analysis shows profound instability at EMD sites [12,16,19,20,21] with both common and distinct copy number variations (CNVs) compared to medullary neoplastic population [12]. Three of the studies that met the criteria in this systematic review declare genomic heterogeneity of EMD due to CNVs, including 1q amplification and 17p deletion [11,12,16]. Indeed, 1q gain is compatible with inferior outcomes in EMD patients, with the number of extra copies being proportionate to prognosis deterioration [22]. Genetic abnormalities of high risk retrieved in extramedullary lesions include t(4;14) [11,20], del (13) [11,19,20], del(1p32) [22,23]. In any case, subclonal CNVs suggest the emergence of new genetic variants, which contribute to treatment resistance and poor outcomes in EMD [12]. In the last few years, the incorporation of gene expression profiling (GEP) in the examination of MM patients with EMD has designated alterations in centrosome genes like CKS1B, YWHAZ, CTBS, NADK [20,24], and NEAT1 [25]. Regarding those genes, when comparing neoplastic cells in extramedullary sites with those in BM milieu, a significant spatial heterogeneity is confirmed [26], suggesting possible association with microenvironment factors.

Neoplastic cells in extramedullary areas succeed in enduring transcriptional changes in pathway-associated genes linked to oxidative stress, glycolysis, proliferation, protein kinase regulator activity, and antigen responses [11,14,20,27]. *TP53* [21], and *RAS* pathway mutations (*KRAS*, *NRAS*) are often identified in EMD patients [23,28,29], suggesting an upregulation of *MAPK* signaling [11], which has been associated with tumor-intrinsic immunosuppression [30]. In addition, deregulation of CXCR4/CXCL12 axon and CCR10 overexpression enables neoplastic cell homing along the extramedullary niche [3,6,31,32]. Notably, Ryu et al. mention that a not so negligible percentage of extramedullary myeloma cases is characterized by aberrant expression of *TRAIL* gene that may activate TRAIL receptors on T-cells, provoking their apoptosis. Along this line of reasoning, expression of major histocompatibility complex (MHC) class I molecule and LIL family protein genes, often reported in EMD, could also deliver suppressive signals to NK-mediated cytotoxicity [14].

Delving further, the methylation profile differs significantly in EMD sites, indicating once more a tendency of clonal evolution of neoplastic cells during BM escape and emergence of EMD. In detail, SHP1 hypermethylation found in MM patients is incriminated for the disease progression by activation of the JAK/STAT3 axon [33,34,35]. In addition, *MYC* translocation seems to play a leading role in neoplastic cell migration from the BM, as it is retrieved in higher percentages in EMD sites than BM [36]. Intriguing also is the fact that, in a single case with paranasal EMD reported by Shi et al., *MYBL2* gene overexpression was associated with activation of the IL-23 signaling pathway, which in turn was related to decreased infiltration of CD8+ cells in the extramedullary niche [37], indicating a definite correlation among genetic profile and IME synthesis in EMD patients.

The installation of an immunosuppressive microenvironment is an established concept in EMD. Overall, our results reveal a prevalence of NK and CD8+ T-cells with an increased expression of PD-1, TIM-3, LAG-3, and TIGIT on their surface in tumor-rich niches. Subsequently, EMD lesions could be considered as “immune-altered” tumors [30] with potential therapeutic implications. Besides dysfunctional CD8+ T-cells, tumor-associated macrophages (CD68+, CD163+, CD86−) and inactivated DCs (CD58−) are also found co-localized with myeloma cells, participating as well in the orchestration of an immunosuppressive state [12,13]. In contrast, as already indicated, activated CD8+ cytotoxic T-cells, M1 macrophages, and DCs with antigen presentation ability (CD58+) are retrieved in tumor-free niches or biopsy margins [12]. This spatial distribution of immune cells along the extramedullary niche possibly suggests that immune cells become inactivated while entering myeloma-rich lesions. The answer to that consequence probably lies either in direct communication between neoplastic and immune cells, or/and in the emergence of a distinct metabolic microenvironment deprived of essential nutrients and amino-acids, a hypothesis well established for solid tumors [38,39]. Finally immune dysregulation is also rendered to the increased number of Tregs and MDSCs found within plasma cell-rich sites [40].

Concerning differences in immune compartment between bone marrow and extramedullary disease, there are prospective avenues yet to be explored. Previously published studies claimed that immune landscape might differ between BM and EMD sites. According to them, immune response may be less regulated in EMD lesions compared to BM niche [41,42]. The results of this systematic review support a more pronounced immunosuppressive microenvironment in EMD sites, with distinct spatial distribution between tumor bed and invasive front [12,13]. On the other hand, limited data suggest a comparable to EMD immune synthesis in BM milieu, consisting of various proportions of T and NK cells, M1 and M2 macrophages, myeloid dendritic cells, and MDSCs [6]. This could be explained by the fact that BM niche is quite dynamic and alters along disease progression. This fact comes in agreement with higher percentages of CD3+ T-cells, reduced Tregs, and decreased expression of immune-checkpoint receptors in CD8+ T-cells in the BM of patients with newly diagnosed MM compared to BM niche of patients with EMD [15,16]. In any case, the findings are limited and rather controversial and require further investigation.

The emerging critical role of the immune microenvironment in EMD places immunotherapy in the center of therapeutic targeting [43]. Along this line, promising results came from the combination of isatuximab (an IgG1 monoclonal antibody directed against CD38), with pomalidomide or carfilzomib (immunomodulatory drugs, IMiDs) and dexamethasone (Isa-Pd, Isa-Kd) [44]. A recent study in patients with soft tissue myeloma detected that those who received either Isa-Pd or Isa-Kd showed prolonged progression-free survival (PFS) and improved overall response rate (ORR) and very good partial response (VGPR) compared to those treated with Pd or Kd alone [45]. The success of the aforementioned findings probably lies in the increase in anti-MM activity induced by the addition of IMiDs to the CD38 monoclonal antibody. In detail, pomalidomide has been shown to enhance CD38 overexpression on regulatory T-cells and increase the isatuximab-mediated effect of antibody-dependent cellular phagocytosis (ADCP), and antibody-dependent cellular cytotoxicity (ADCC) [44]. In addition, isatuximab can directly activate NK-mediated cytotoxicity via crosslinking of CD38 and CD16 or enhance T- cell mediated immune response [46]. On the other hand, daratumumab, another IgG κ monoclonal antibody with a distinct epitope on CD38 [47,48], showed limited efficacy in a cohort of 41 patients with EMD [49], possibly due to the lack of combination with IMiD as well as to the distinct mode of action compared to isatuximab. Specifically, daratumumab is not capable of causing cell death without the aid of crosslinking agents, nor modifying CD38 enzymatic activity [50]. However, further study is required to understand the immune contexture of EMD and its influence on response to therapy, in order to optimize the multitarget treatment for EMD.

## 5. Study Limitations

The prime limitation of the systematic review presented herein is the relatively small number of studies that met our inclusion criteria. This could affect the validity of our results but also highlights the necessity for further examination. In addition, the small sample size of each research study impacts the precision of our findings. To be mentioned, the selected publications varied in terms of study design, follow-up period, and methodologies applied for both myeloma cells and microenvironment landscape rendering difficulties when comparing and rarely leading to controversies.

## 6. Conclusions

Tumors are ecosystems, and so it seems to be the case for MM, with continuous evolution of clones, some of which gain aggressive properties and dynamic to spread to extramedullary sites. This continuous process gives rise to intra- and inter- genetic variations, raising almost impregnable walls for therapeutic interventions. EMD, as an end-stage station of disease progression, is characterized by greater genomic variability and more pronounced immunosuppression related to medullary disease. Spatial and temporal heterogeneity is also noted in IME of EMD, implying a possible correlation of the former with the distinct molecular profile. Indeed, the emergence of an “immune-altered” landscape rationally reflects genetic and/or transcriptional alterations on signaling pathways related to proliferation, “homing”, and cell migration. However, no molecular event-specific immune contexture has been identified from this systematic review, possibly due to the relatively small number of cases studied, as well as the potential impact of local adaptive processes apart from the tumor-intrinsic ones. Focusing on the molecular pathways and genetic abnormalities that correlate with specific microenvironment or its heterogeneity across extramedullary milieu offers potential to design personalized multi-targeted treatment strategies and overcome resistance to current therapeutic modalities.

## Figures and Tables

**Table 1:** Summary of studies investigating IME in EMD.

Authors, Year [Ref.]	Number of Patients with EMD	Sex Male: Female	Median Age (Years)	Number of Samples	Methodology for MM Cells	Methodology for IME	Main Results in Regard to EMD
Jelinek et al., 2024 [11]	14	10:4	59	14	FISH, WES	Bulk RNA-seq, scRNA-seq, FC	EMD cells have a higher frequency of 1q21 gain, mutations in the MAPK pathway, activation of proliferation-associated pathways and decreased homing to BM. EMD IME is mainly composed of CD8+ and NK cells.
John et al., 2024 [12]	11	7:4	61	14	Spatial transcriptome analysis and scRNA-seq	Spatial transcriptome analysis and scRNA-seq	Intratumor heterogeneity and genetic instability of MM cells. Differences in spatial distribution between dysfunctional/exhausted and activated T-cells, as well as M1 and M2, DC1 and DC2.
Qi et al., 2024 [13]	31	17:14	55	16	None	Multiplex IF	Differences in spatial distribution of exhausted CD8+ T-cell subsets and M2 macrophages. Immune suppressor cells may create a hostile IME and mitigate the CAR T-cell activity.
Ryu et al., 2020 [14]	5	3:2	51	5	scRNA-seq	scRNA-seq	Distinct transcriptional characteristics of MM cells in EMD compared to BM. Immune evasion due to cytotoxic-exhausted phenotype in EMD.
Zhang et al., 2024 [15]	9	7:2	53	6	Multicolored fluorescent FC	Multicolored fluorescent FC	No differences in Galectin-9 and CD155 expression on myeloma cells among PB, BM, and EMD tissue. Lower CD3+ cells and exhausted T-cell phenotype characterized among PB, BM, and EMD tissue. Lower CD3+ cells and exhausted T-cell phenotype characterized by higher TIGIT and TIM-3 expression levels on CD8+ cells in EMD.
Merz et al., 2023 [16]	4	3:1	NA	4	FISH, WES, IF	TCR-seq, FISH, WES, IF	Significant differences in chromosomal aberrations and gene mutations between intra- and extramedulary disease. Immune evasion due to increased Tregs and MDSCs in EMD after induction therapy.

Abbreviations: EMD: extramedullary disease; IME: immune microenvironment: MM: multiple myeloma; scRNA-seq: Single-cell RNA sequencing; NA: Not assessed; IF: immunofluorescence; FISH: Fluorescent in situ hybridization; WES: Whole Exome Sequencing; TCR-seq: TCR sequencing; Tregs: T-regulatory cells; MDSCs: myeloid-derived suppressor cells; FC: flow cytometry; BM: bone marrow; NK: natural killer; PB: peripheral blood; TIGIT: T-cell immunoreceptor with immunoglobulin and ITIM domain; TIM-3: T-cell Ig- and mucin-domain-containing molecule-3; CAR: chimeric antigen receptor; DC: dendritic cells.

## Data Availability

No new data were created or analyzed in this systematic review. Data sharing is not applicable to this article.

## Supplementary Materials

The following supporting information can be downloaded at: https://www.mdpi.com/article/10.3390/cancers17071081/s1, Figure S1: Risk of Bias assessment using ROBINS-E tool.
