# Supplementary figures and images for: Unveiling Extramedullary Myeloma Immune Microenvironment: A Systematic Review
Source: Cancers (Basel). 2025 Mar 24;17(7):1081. doi: 10.3390/cancers17071081 (PMC11987838; doi:10.3390/cancers17071081)

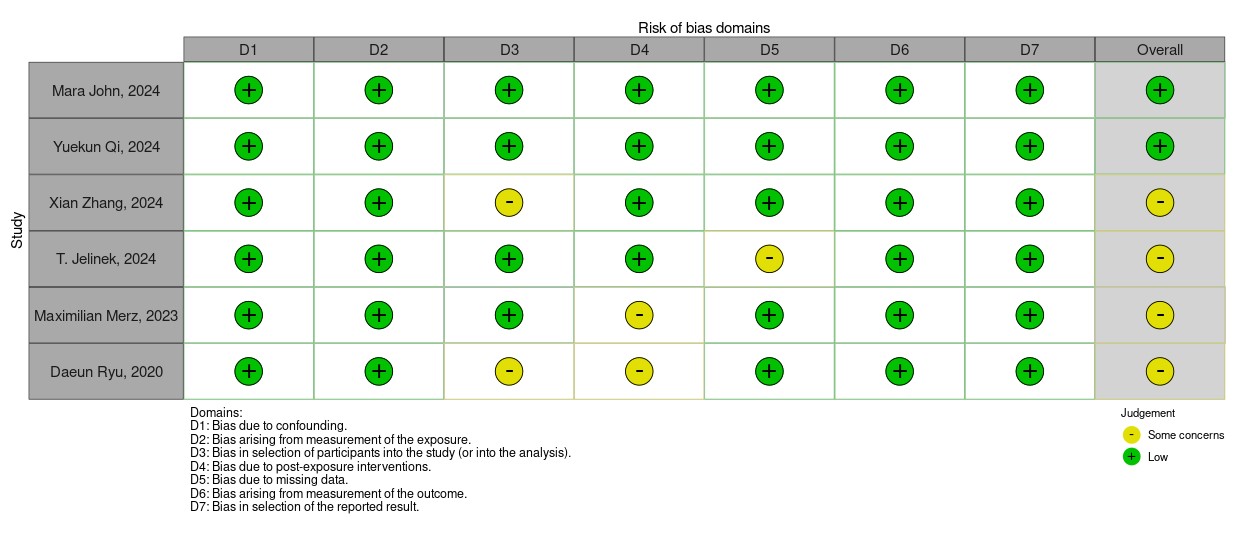

Supplement: Supplementary file 1 [file cancers-17-01081-s001.zip › cancers-3482116-supplementary.jpg]
